# Modulation of colonic immunometabolic responses during *Clostridioides difficile* infection ameliorates disease severity and inflammation

**DOI:** 10.1038/s41598-023-41847-2

**Published:** 2023-09-07

**Authors:** Nuria Tubau-Juni, Josep Bassaganya-Riera, Andrew J. Leber, Sameeksha S. Alva, Ryan Baker, Raquel Hontecillas

**Affiliations:** NIMML Institute, Blacksburg, VA 24060 USA

**Keywords:** Clostridium difficile, Mucosal immunology

## Abstract

*Clostridioides difficile* infection (CDI) is the leading cause of antibiotic-associated diarrhea, and its clinical symptoms can span from asymptomatic colonization to pseudomembranous colitis and even death. The current standard of care for CDI is antibiotic treatment to achieve bacterial clearance; however, 15 to 35% of patients experience recurrence after initial response to antibiotics. We have conducted a comprehensive, global colonic transcriptomics analysis of a 10-day study in mice to provide new insights on the local host response during CDI and identify novel host metabolic mechanisms with therapeutic potential. The analysis indicates major alterations of colonic gene expression kinetics at the acute infection stage, that are restored during the recovery phase. At the metabolic level, we observe a biphasic response pattern characterized by upregulated glycolytic metabolism during the peak of inflammation, while mitochondrial metabolism predominates during the recovery/healing stage. Inhibition of glycolysis via 2-Deoxy-d-glucose (2-DG) administration during CDI decreases disease severity, protects from mortality, and ameliorates colitis in vivo. Additionally, 2-DG also protects intestinal epithelial cells from *C. difficile* toxin damage, preventing loss of barrier integrity and secretion of proinflammatory mediators. These data postulate the pharmacological targeting of host immunometabolic pathways as novel treatment modalities for CDI.

## Introduction

*Clostridioides difficile* is a gram-positive, spore-forming, anaerobic bacterium that colonizes the gastrointestinal (GI) tract^1^. *C. difficile* infection (CDI) is the leading cause of antibiotic-associated diarrhea, responsible for 15–25% of cases^2^, and clinical symptoms can vary from asymptomatic colonization to severe diarrhea, pseudomembranous colitis and death^3^. As reported by the Centers for Disease Control (CDC), almost half a million cases of CDI occur yearly only in the U.S., with about 29,000 patients with fatal outcomes within the first month of diagnosis^4^. *C. difficile* is present in the gut microbiome of an estimated 5% of adults and 15–70% of children, and the prevalence significantly increases in hospitalized patients and the elderly^5^. In homeostatic conditions, commensal gut microbiome species prevent the germination and overgrowth of ingested *C. difficile* spores, however, upon disruption of the gut microbiome due to antibiotic administration, intestinal colonization resistance is reduced, *C. difficile* proliferates and establishes infection^3, 6^. Paradoxically the standard of care (SOC) of CDI patients is the treatment with more antibiotics, that can further disrupt the commensal gut microbiome and lead to recurrence^7^. Indeed, one of the main challenges of managing CDI is the high recurrence rate, estimated to be between 15 and 35% after an initial positive response to treatment^8^.

*C. difficile* is an extracellular pathogen and its virulence is mainly attributed to secreted enzymes and toxins^9^. *C. difficile* toxin A (TcdA) and B (TcdB) are the two major virulence factors^10^. Once internalized, *C. difficile* toxins inactivate small GTPases, including members from the Rho, Rac and Cdc42 families. Enterocytes are the first line of protection in the GI tract, and the main cellular target of *C. difficile* toxins. Exposure to TcdA and TcdB results in alteration of cytoskeletal proteins, cell rounding, loss of tight junctions and cell death, that ultimately lead to impaired epithelial barrier function, with fluid secretion and bacterial translocation^11, 12^. Toxin-mediated cell damage and the subsequent bacterial translocation lead to the initiation of a strong immune response characterized by the secretion of proinflammatory cytokines and chemokines, i.e. IL-8, IL-1β, epithelial cell production of antimicrobial peptides (AMP) and recruitment of inflammatory cells to the site of infection^13^. Indeed, during the acute inflammatory phase, neutrophilic infiltration of the colonic mucosa upon IL-8 secretion is one of the earliest and key features of CDI^14, 15^. Th17 effector responses are also enhanced during the effector period, as well as mucosal infiltration of other innate immune cells (i.e. mast cells, monocytes, etc.)^13^, while regulatory T cells (Treg) are dominant in the disease recovery phase during colonic tissue resolution^16^.

Cellular metabolism helps fulfill the energetic demands of the cell for generating biosynthetic precursors, and it is a critical regulator of cell phenotype and function^17^. The colonic mucosa is composed of an epithelial layer that creates a barrier, and the underlying lamina propria which is enriched in immune cells to provide protection against invading organisms. In intestinal epithelial cells, the overall metabolic preferences switch during maturation and differentiation along the crypt to villus axis^18, 19^. In immune cells, upon initiation of inflammatory responses, effector cells undergo a metabolic reprogramming characterized by increased anaerobic glucose metabolism and lactate production, to facilitate the quick execution of effector responses, while quiescent and regulatory cells (Treg) favor mitochondrial respiration and oxidative metabolism to efficiently generate energy^20, 21^. Similarly, terminally differentiated colonocytes that rely on mitochondrial β-oxidation of fatty acids in homeostatic conditions, undergo a metabolic shift if stimulated by inflammatory mediators towards anaerobic glycolysis^22^. Thus, colonic metabolic changes are closely correlated with functional and phenotypic changes in immune and epithelial cells.

In this study, we conducted a comprehensive, systems-wide bioinformatics analysis and in silico metabolic modeling from a high-resolution longitudinal time course global transcriptomics dataset from CDI to characterize novel host immunometabolic mechanisms with therapeutic potential. The bioinformatics analysis indicated a two-stage host metabolic modulation at the colon during CDI characterized by upregulated glycolysis at the peak of inflammation, while mitochondrial metabolism was enriched during the disease recovery and tissue resolution phase. To validate these bioinformatics predictions related to metabolic reprogramming in vivo, we determined the effects of inhibition of glycolysis via oral 2-Deoxy-D-glucose administration on disease severity, mortality, and colonic inflammation in a mouse model of CDI. To provide in vitro validation in epithelial cells, we examined whether inhibiting glycolytic pathways in intestinal epithelial cells would provide protection from *C. difficile* toxin damage, preventing loss of barrier integrity and secretion of proinflammatory cytokines. Our data, postulates the modulation of host mucosal immunometabolic responses as a novel therapeutic approach for the treatment of CDI, and a safer and more sustainable alternative to antimicrobial treatment.

## Results

### Global transcriptomics analysis of colonic host responses during CDI

**Figure 1:**
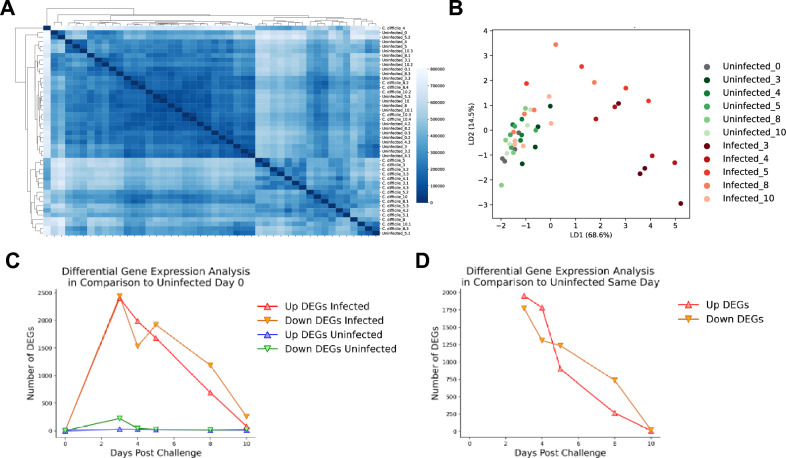
Global gene expression analysis of 10-day RNA-seq study of colonic host responses in *C. difficile* infection (CDI). Global transcriptomic sequencing was conducted on colonic RNA extracted at day 0, 3, 4, 5, 8 and 10 post *C. difficile* challenge from infected and uninfected WT mice. Hierarchical clustering (**A**) for a given sample in the data set. Linear discriminant analysis (**B**) for samples in the dataset. Number of differentially expressed genes (DEGs) for each experimental group in comparison to uninfected on day 0 (**C**). Number of DEGs between uninfected and infected experimental groupings for each given day (**D**). (n = 4–5).

We first sought to explore the dynamics of the host response during *Clostridioides difficile* infection (CDI) to gain a better understanding of the mucosal responses during the infection and with the goal of identifying novel mechanisms with therapeutic potential for the treatment of CDI. We conducted a 10-day longitudinal time course RNA-Seq study of colons extracted from mice infected with *C. difficile* and uninfected controls to establish differentially expressed host genes and clusters sharing similar expression patterns. Samples were collected at days 0, 3, 4, 5, 8 and 10 post-infection to encompass the distinctive phases of active disease and recovery during CDI. Both infected and uninfected groups were subjected to prior antibiotic regime. Quality control analysis of the RNA-Seq study indicated > 81% of all filtered paired genes for a given sample mapped to the mouse genome except for three samples with > 73%. Hierarchical clustering (Fig. 1A) and linear discriminant analysis (Fig. 1B) indicated very tight grouping for a large subset of the various experimental cohorts, while a smaller subset, representing the samples from infected mice on days 3 and 4, and some from day 5, were distinct. In this model, days 3 and 4 correspond to the peak of inflammation. Differential gene expression analysis identified days 3, 4 and 5 post infection as the days with higher number of differentially expressed genes (DEGs) (Supplementary Fig. 1A). Comparison of the *C. difficile*-infected and uninfected groups to the uninfected day 0 indicated that the antibiotic treatment alone has a slight impact in gene expression although a significantly larger number of genes were differentially expressed in the infected group (Fig. 1C). In this group, the highest number of DEGs was observed at day 3 post infection (~ 2500 genes), then, DEGs started to decrease (> 1500 genes at day 4 and 5) reaching considerably lower numbers at day 8 and minimal levels at day 10, at the end of the recovery phase, mimicking the control group. We then sought to identify DEGs specific to infection by performing pairwise comparisons on each day. The maximum number of DEGs occurred on days 3 and 4 (> 1750 upregulated genes), began decreasing on day 4, and 5 for downregulated genes, and resolved by day 10 post infection (Fig. 1D). Note that a Wilks test for time and infection reported a total of 6797 DEGs (*P* < 0.05). This data indicated that with this experimental design, both the early infection phase and the later recovery phase were covered. Overall, this initial global transcriptomics analysis indicated that colonic gene expression kinetics is significantly altered during CDI, particularly during the peak of inflammation, displaying a very distinctive expression profile in the two phases of the disease.

### CDI alters host metabolism in a biphasic manner

**Figure 2:**
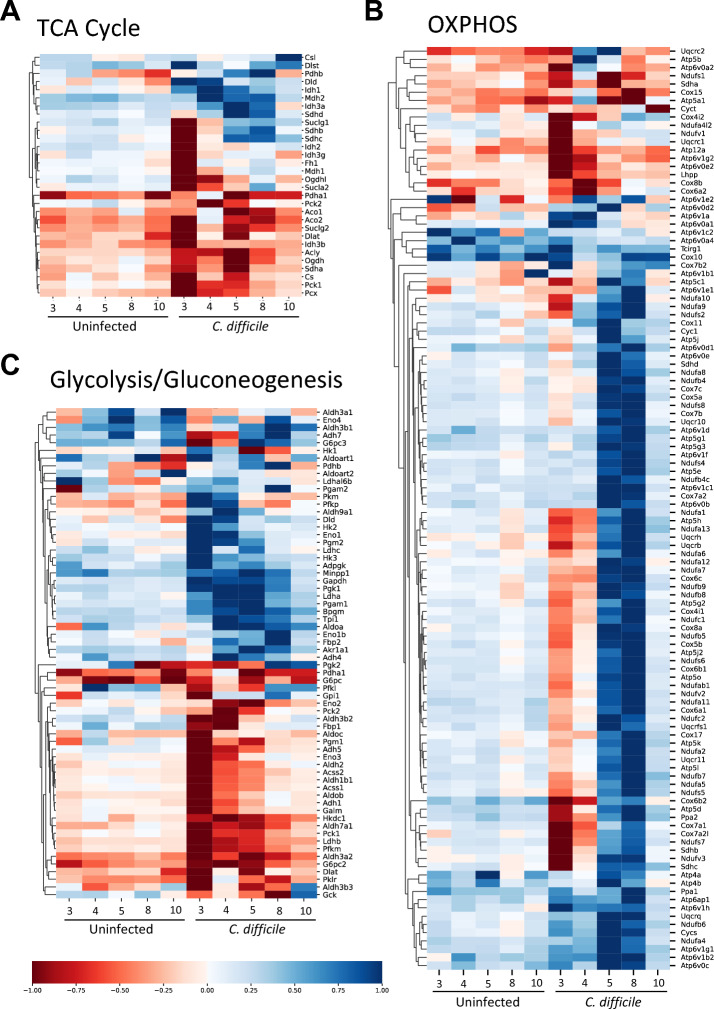
Host gene expression responses to CDI enrich metabolic processes with a distinctive peak of inflammation versus recovery phase patterns*.* RNA-seq data from each given day of the 10-day dataset (day 0, 3, 4, 5, 8 and 10) from colonic *C. difficile*-infected and uninfected samples was normalized to uninfected day 0 and displayed as fold change. Each row was normalized such that the greatest log change was mapped to either 1 or -1 depending on the sign of the change. RNA-seq data was filtered to only include the genes associated with each KEGG pathway. Heat map of Hierarchically clustered genes associated with TCA cycle (**A**), the Oxidative phosphorylation (**B**), and glycolysis/gluconeogenesis (**C**) are displayed.

Enrichment analysis comparing uninfected vs. infected groups identified many pathways enriched due to infection (P < 0.05, FDR). Of note was the enrichment in multiple metabolic pathways, including carbohydrate metabolism, including both glycolysis/gluconeogenesis and the TCA cycle (Supplementary Fig. 2). Given these results and the reported role of cell metabolism in regulating intestinal epithelium function, we decided to focus our analysis on host mucosal metabolism during CDI. To gain a global understanding of the changes in metabolic status in the colon during infection, we generated hierarchical clustering heatmaps of three metabolic pathways (TCA cycle, oxidative phosphorylation, and glycolysis/gluconeogenesis) using filtered RNA-seq data. Our results indicated that over 70% of the TCA cycle associated genes were downregulated during the acute phase of the infection, particularly at day 3 post infection, but also on dpi 4 and 5 (> 55%) (Fig. 2A; Supplementary Fig. 3). In correlation to the TCA cycle genes, the oxidative phosphorylation (OXPHOS) pathway was also enriched by genes decreasing in expression during the peak of inflammation, however, at day post infection 5 and 8, during the recovery, there was a dramatic upregulation of over 75% of the genes within the pathway. Note that by dpi 10, expression levels resembled the ones in the uninfected group (Fig. 2B; Supplementary Fig. 4). Interestingly, gene expression analysis of glycolysis and gluconeogenesis reported two distinctive opposite patterns, with approximately 50% of the genes displaying highly upregulated expression at dpi 3 and 4, and less pronounced at dpi 5, while the other half were downregulated during the same time points (Fig. 2C; Supplementary Fig. 5). However, focusing into the specific genes included in each cluster, the first group (upregulated expression during acute inflammation) is enriched by genes encoding enzymes directly involved in the reactions responsible for the breakdown of glucose into lactate, including the *Hk* and *Pkm*, encoding the enzymes responsible of the first and third limiting steps of glycolysis, respectively, and *Ldha*, that converts pyruvate to lactate. In contrast, the second group (characterized by genes with downregulated expression during the acute phase) is enriched by gluconeogenic genes or genes favoring oxidative metabolism, including *Pck*, *G6pc* and *Fbp*, three genes from early or late stages of gluconeogenesis, and *Ldhb*, and *Pdhb*, that encode enzymes that promote pyruvate formation and pyruvate entrance to the TCA cycle, respectively. Thus, this indicates upregulation of glycolytic metabolism during the peak of the inflammation, while the gluconeogenic pathway is suppressed. In conclusion, this metabolic bioinformatics analysis indicates a significant change in the activity of host metabolic pathways during CDI, that is correlated to the distinctive phases of the disease and highlights an increase of anaerobic metabolism during the peak of inflammation, while mitochondrial metabolism is favored during the recovery phase.

**Figure 3:**
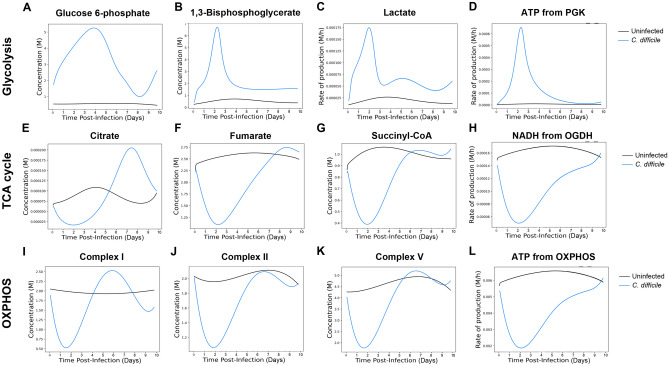
M^2^ pipeline simulation to colonic host responses to CDI. Concentration of glycolytic metabolites Glucose 6-phosphate (G6-P, **A**) and 1,3-Bisphosphoglycerate (**B**, 1,3BPG). Rate of production of Lactate (**C**) and ATP from phosphoglycerate kinase (PGK) reaction (**D**). Concentration of TCA cycle metabolites Citrate (**E**), Fumarate (**F**) and Succinyl-CoA (**G**), and rate of production of NADH from oxoglutarate dehydrogenase (OGDH) reaction (**H**). Concentration of Complex I (**I**), Complex II (**J**) and Complex V (**K**) from the chain of electrons, and rate of production of ATP from OXPHOS (**L**).

To further characterize the metabolic changes caused during CDI, we utilized our previously developed M^2^ pipeline^23^, that uses transcriptomic datasets and integrates them in an ODE-based model^24^ for time course simulations of metabolic pathways. The 10-day time course RNA-seq data was imported to the M^2^ pipeline to model changes in glycolysis, TCA cycle and OXPHOS during CDI. The results indicate a peak of glycolytic activity during the initial phase of the infection, while no major changes were observed in the uninfected group. Indeed, simulated concentrations of the glycolytic metabolites G6-P (Fig. 3A) and 1,3BPG (Fig. 3B), generated by HK and GAPDH reactions, respectively, plus rate of production of lactate (Fig. 3C) and ATP production from glycolytic PGK (Fig. 3D) are highly upregulated upon infection and return to basal levels during the recovery phase. Regarding the TCA cycle metabolites assessed, simulated concentrations of citrate (Fig. 3E), fumarate (Fig. 3F) and succinyl-CoA (Fig. 3G) display an opposite pattern, characterized by downregulation during the peak of inflammation, while their concentration levels increase during the recovery phase. Note that rate of production of NADH from OGDH reaction (Fig. 3H) also displays a similar trend. Accordingly, simulated concentrations of Complex I (Fig. 3I), Complex II (Fig. 3J) and Complex V (Fig. 3K) of the electron transport chain, plus ATP production from OXPHOS (Fig. 3L) are also suppressed during the acute infection stage, while levels are later rescued during the recovery. Therefore, the M^2^ pipeline predictions support the initial bioinformatics assessment, and pinpoint the biphasic metabolic host response during CDI consisting of an initial enhancement of glycolytic metabolism during the acute inflammatory phase, while mitochondrial pathways are favored during the later induction of regulatory and tissue healing responses. Simulated enzymatic concentration plus calibration experimental data points corresponding to the metabolites presented can be found in Supplementary Fig. 6.

### In vivo validation of *C. difficile*-induced host metabolic changes

**Figure 4:**
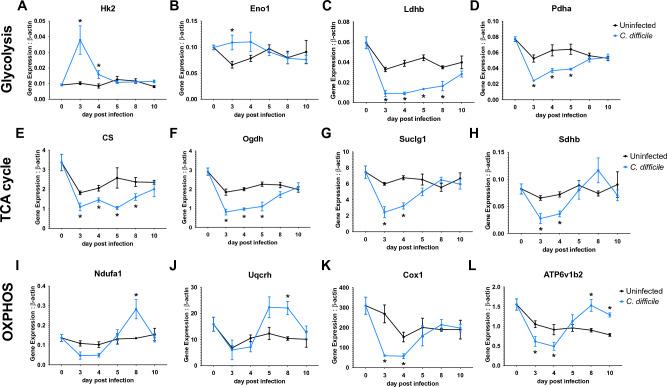
Gene expression validation of metabolic analysis in colonic tissue*.* Validated gene expression through qRT-PCR of glycolytic genes Hexokinase 2 (**A,** Hk2), Enolase 1 (**B**, Eno 1), Lactate dehydrogenase b (**C**, Ldhb), and, Pyruvate dehydrogenase a (**D**, Pdha); TCA cycle genes Citrate synthetase (**E**, CS), Oxoglutarate dehydrogenase (**F**, Ogdh), Succinate-CoA Ligase GDP/ADP-Forming Subunit Alpha (**G**, Suclg1), and, Succinate dehydrogenase Complex Iron Sulfur Subunit B (**H**, Sdhb); and oxidative phosphorylation (OXPHOS) genes NADH:Ubiquinone Oxidoreductase Subunit A1 (**I**, Ndufa 1), Ubiquinol-Cytochrome C Reductase, Complex III Subunit VIII (**J**, Uqcrh), Cytochrome C Oxidase Subunit 1 (**K**, Cox1), and ATPase H + transporting V1 subunit B1 (**L**, Atp6v1b1) in colonic tissue from *C. difficile*-infected and uninfected mice at dpi 0, 3, 4, 5, 8 and 10. Data expressed as mean ± SEM (n = 4–5). Asterisks mark statistical significance. *P ≤ 0.05.

To confirm the results obtained in this comprehensive systems-wide bioinformatic analysis and M^2^ pipeline simulations, the expression of several key metabolic genes from the three metabolic pathways was validated through qRT-PCR. Consistent with the RNA-seq results, glycolytic *Hk2* (Fig. 4A) and *Eno1* (Fig. 4B) displayed upregulated expression at day 3 during the peak of inflammation in the infected group and returned to baseline at day 5, while the expression of *Ldhb* (Fig. 4C) and *Pdha* (Fig. 4D) was downregulated. Additionally, the TCA cycle genes assessed presented the described decrease of expression starting at day post infection 3 and continued downregulated up to day post infection 8, including *CS* (Fig. 4E), *Ogdh* (Fig. 4F), and *Suclg1* (Fig. 4G) or day post infection 4, as observed in *Sdhb* (Fig. 4H). Regarding the oxidative phosphorylation genes analyzed, *Ndufa1* (Fig. 4I) and *ATP6v1b2* (Fig. 4L**)** displayed the reported kinetics, characterized by an initial decrease of expression during days 3 and 4 post infection, followed by an increase during the recovery phase. *Uqcrh* (Fig. 4J) presented a delayed upregulated response, while *Cox1* (Fig. 4K) displayed the earlier decrease of expression.

**Figure 5:**
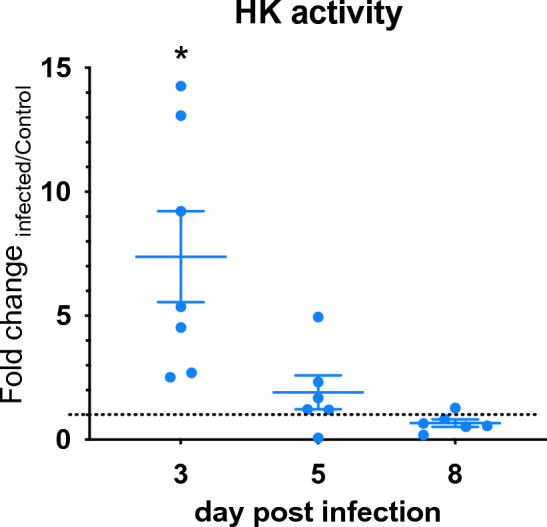
Functional validation of gene expression metabolic changes in vivo. WT mice were infected with *C. difficile* and samples collected at dpi 3, 5, and 8. Uninfected controls were also included. Colonic HK activity was measured using an enzymatic assay kit. Data expressed as mean ± SEM (n = 6–7). Asterisks mark statistical significance. *P ≤ 0.05.

To validate whether the metabolic changes identified at the gene expression level were translated functionally, enzymatic activity of HK, the first limiting enzyme of the glycolysis, was quantified in colonic tissue of infected and uninfected mice at dpi 3, 5 and 8. In correlation to the gene expression results, our data reported an over sevenfold increase of HK activity at day 3 post infection in the infected group compared to the uninfected control (Fig. 5). At days 5 and 8 post infection, HK activity significantly decreased in the infected groups and no statistical differences were reported compared to the control.

Together, the bioinformatics and computational analysis plus the experimental validation indicate an increase of anaerobic metabolism during the peak of inflammation, at days 3 and 4, with a concomitant downregulation of oxidative pathways. Conversely, during the recovery phase, glucose metabolism is suppressed, and mitochondrial respiration is favored, with increased expression of TCA cycle and OXPHOS genes.

### Glycolytic inhibition ameliorates disease severity and decreases colonic inflammation in a mouse model of CDI

**Figure 6:**
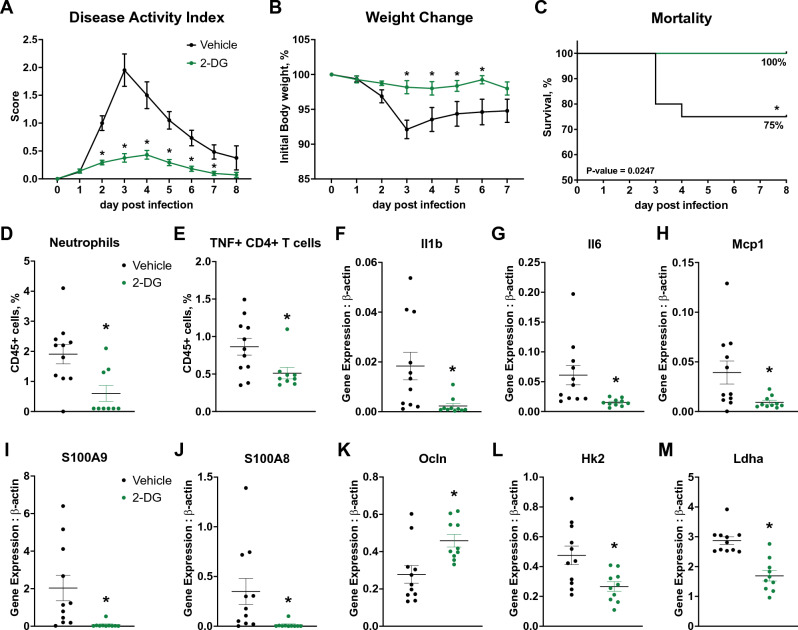
Efficacy of 2-DG in a mouse model of CDI. WT mice were infected with *C. difficile* and treated with oral 2-DG (500 mg/kg/d) daily starting at dpi 1. Disease Activity index (**A**), weight loss (**B**) and mortality rate (**C**) were monitored daily for a period of 8 days. Colons were collected at dpi4 and the proportion of infiltrating neutrophils (**D**) and TNF + CD4 + T cells (**E**) were quantified via flow cytometry. Colonic expression of *Il1b* (**F**), *Il6* (**G**), *Mcp1* (**H**) *S100A9* (**I**), *S100A8* (**J**), *Ocln* (**K**), *Hk2* (**L**) and *Ldha* (**M**) was assessed through qRT-PCR. Data expressed as mean ± SEM (n = 9–20). Asterisks mark statistical significance. *P ≤ 0.05.

Given the upregulated glycolytic metabolism during the inflammatory phase, together with the validated increase in HK activity upon infection, we sought to explore the effects of 2-DG on CDI. 2-DG is a modified glucose molecule in which the 2-hydroxyl group has been replaced by hydrogen, preventing its progression through glycolysis. 2-DG competes with glucose to bind HK and inhibits glycolysis. C57BL/6 WT mice were infected with *C. difficile* VPI 10463 and monitored for a period of 8 days. 2-DG treatment (500 mg/kg/day) was orally administered daily through orogastric gavage starting at dpi 1. Oral 2-DG treatment ameliorated disease severity (Fig. 6A) and prevented loss of initial body weight (Fig. 6B), in comparison to the group treated with vehicle. Moreover, oral 2-DG improved the survival rates of infected mice, with 100% survival in *C. difficile*-infected mice that received 2-DG, vs 75% survival in the *C. difficile*-infected mice that received vehicle only (Fig. 6C). Inhibition of glycolytic metabolism in infected mice resulted in reduced infiltration of neutrophils (Fig. 6D) and TNF-secreting CD4+ T cells (Fig. 6E) at the colonic tissue plus suppressed expression of proinflammatory mediators *Il1b* (Fig. 6F), *Il6* (Fig. 6G) and *mcp1* (Fig. 6H). Antimicrobial peptide production was also greatly reduced in the 2-DG treated group, for both *S100A9* (Fig. 6I) and *S100A8* (Fig. 6J), while the expression of the tight junction gene *Ocln* (Fig. 6K) was increased. Additionally, 2-DG-treated mice displayed downregulated expression of both *Hk2* (Fig. 6L) and *Ldha* (Fig. 6M) glycolytic enzymes compared to the vehicle control group. To confirm that the observed effects of 2-DG were due to targeting host responses, and not to direct modulation of *C. difficile* growth, *C. difficile* was cultured in vitro in anaerobic meat chopped tubes injected with 2-DG at 1, 10 and 100 μg/mL or vehicle for 8 h. Our data reported no differences between the 2-DG treatment groups or the vehicle control (Supplementary Fig. 5). Notably, vancomycin treatment suppressed bacterial growth by tenfold (Supplementary Fig. 5).

### Inhibition of glycolytic metabolism decreases epithelial cell permeability and secretion of inflammatory mediators upon exposure to TcdA and TcdB

**Figure 7:**
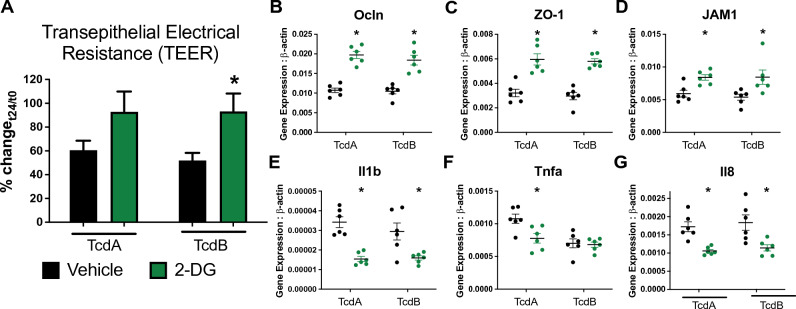
Effects of 2-DG treatment in T84 cells exposed to TcdA or TcdB. T84 cells were challenged with 100 or 250 ng/mL of TcdA or TcdB, treated with 10 mM of 2-DG and cultured for 24 h. Transepithelial electrical resistance (TEER) (**A**) was quantified. Expression of tight junction genes Ocludin (**B**, Ocln), Tight junction protein 1 (**C**, ZO-1), F11 receptor (**D**, JAM1), and proinflammatory cytokines *Il1b* (**E**), *Tnfa* (**F**) and *Il8* (**G**) was assessed through qRT-PCR. Data expressed as mean ± SEM (n = 6–12). Asterisks mark statistical significance.*P ≤ 0.05.

The epithelial barrier is the first line of protection against enteric pathogenic infections. Based on the positive effects reported by 2-DG administration in an in vivo model of CDI, we sought to explore the potential effects of inhibiting glycolysis in intestinal epithelial cells exposed to *C. difficile* toxins in vitro. T84 cells were exposed to TcdA or TcdB and treated with 10 mM 2-DG for 24 h. Treatment with 2-DG resulted in increased transepithelial electrical resistance (TEER) upon exposure to both TcdA and TcdB compared to the vehicle control (Fig. 7A**)**. Interestingly, 2-DG treatment of T84 cells not exposed to TcdA or TcdB did not induce changes in TEER compared to the vehicle control group and the baseline timepoint (Supplementary Fig. 6). Consistent with the decreased epithelial cell permeability, expression of tight junction genes Ocln (Fig. 7B), ZO-1 (Fig. 7C) and JAM1 (Fig. 7D) were upregulated in the 2-DG treated group. Immunologically, inhibition of glycolysis resulted in reduced expression of proinflammatory mediators, as observed in vivo. Indeed, expression of *Il1b* (Fig. 7E), *Tnfa* (Fig. 7F**,** upon TcdA exposure) and *Il8* (Fig. 7G) were suppressed in the 2-DG group compared to the untreated control.

## Discussion

This study presents a comprehensive, systems-wide transcriptomics analysis of host immunometabolic responses to CDI in mice. Our analysis identified a metabolic reprogramming during CDI at the colonic level that shifts between the peak of inflammation and the disease recovery and tissue resolution/healing phase. We performed follow up validation studies to determine whether the pharmacological manipulation of these metabolic responses by blocking glycolytic pathways has therapeutic benefits. In vivo and in vitro validation of the RNA-seq findings confirmed the critical role of cellular metabolism during host response to CDI. Indeed, inhibition of glycolytic metabolism through oral 2-Deoxy-D-glucose (2-DG) administration ameliorated disease severity and colonic inflammation in vivo while protecting epithelial cells from *C. difficile* toxin-associated damage in vitro.

Colonic epithelial cells are the main targets of *C. difficile* toxins and inflamed epithelial cells are a key component in the pathogenesis of the disease. In the initial phases of infection, TcdA and TcdB directly disrupt tight junctions and the cytoskeleton structure of intestinal epithelial cells (IEC), by inducing redistribution of OCLN, ZO-1, and actin filaments, resulting in increased epithelial cell permeability, intestinal bacterial translocation, and induction of a potent immunoinflammatory response^25–27^. Extensive toxin-mediated epithelial cell damage can result in impaired stem cell compartment at the crypts, interfering with the ability of the epithelium to regenerate, and therefore, impairing tissue healing and disease recovery following infection^28^. CDI also induces goblet cell loss^29^, and MUC2 has been identified as an important player protecting epithelial cells from toxin-mediated damage^30^. Immunologically, toxin-damaged epithelial cells play a key role in inducing effector immune responses to CDI through secretion of proinflammatory cytokines and chemokines such as IL-8, IL-1b. Indeed, during CDI, inflamed intestinal epithelial cells induce IL-8-mediated mucosal infiltration of neutrophils. Interestingly, fecal IL-8^35^ and IL-1b^31, 32^, depth of colonic epithelium damage, and toxin titer^28^, but not pathogen loads, correlate more closely with disease severity^31^. These data postulate that targeting host responses at the intestinal epithelial barrier to preserve its integrity and recover GI homeostasis might be a promising therapeutic alternative to the current indiscriminate antimicrobial therapy.

The metabolic profiles of immune and intestinal epithelial cells are tightly correlated with their phenotype and function. Our results indicate an increase of anaerobic glycolysis at days 3 and 4 post CDI, coinciding with the disruption of the epithelial barrier and infiltration of the colonic mucosa by effector immune cell subsets, while oxidative metabolism is upregulated during the recovery phase, when regulatory T cells (Treg) and tissue healing functions predominate. Importantly, this biphasic immunometabolic host response correlates with the predictions of our advanced computational model of bioenergetic mechanisms that modulate CD4+ T cell functions, that reported increased lactate concentration in the peak of inflammation, while ATP generated from OXPHOS is suppressed^24^. Alteration of host metabolism in intestinal epithelial cells has also been reported during enteric infection of other extracellular pathogens^33^. For instance, infection with *Citrobacter rodentium*, that models human infection with Enteropathogenic and enterohaemorrhagic *Escherichia coli* (EPEC and EHEC) results in mitochondrial disfunction, and downregulation of gluconeogenesis, lipid metabolism, TCA cycle and OXPHOS, while aerobic glycolysis and cholesterol metabolism are favored^34–36^. Additionally, metabolomic analyses from Enterotoxigenic *E. coli* (ETEC) infection reports the alteration of several metabolic pathways upon infection^37, 38^, suggesting also the increase in glycolysis and PPP, while urea cycle is suppressed^33^. Interestingly, mitochondrial metabolism and function regulates intestinal stem cell programming, regulating epithelial cell differentiation, proliferation, and renewal, plus the proliferation and activation of cells involved in wound-healing in intestinal epithelium^18, 39, 40^.

We report novel evidence that exogenous inhibition of glycolysis through 2-DG treatment of intestinal epithelial cells exposed to *C. difficile* toxins decreases cell permeability while upregulating the expression of several tight junction genes, including Ocln and ZO-1. AMP-activated protein kinase (AMPK) is a metabolic regulator that is activated in response to depletion of ATP upon cellular stress, also involved in metabolic balance^41^. AMPK facilitates tight junction assembly in epithelial cells, resulting in increased ZO-1 redistribution and decreasing cell permeability^42^. This suggests that enhanced barrier function upon modulation of metabolic preferences in intoxicated cells might be mediated through AMPK activation. Indeed, treatment of unpolarized cells with 2-DG results in generation of a polarized actin cytoskeleton and brush-border like structure trough AMPK activation^43^. Moreover, in vivo inhibition of glycolysis during CDI protects from mortality while decreasing exacerbated inflammatory and effector responses and increases expression of tight junction gene Ocln. Of note, despite that low levels of glucose might slightly upregulate toxin production^44^, high levels (0.5–1%) suppress the production of toxin^44–47^, suggesting that the potential 2-DG-mediated inhibition of *C. difficile* hexokinase will likely not decrease toxin production. Additionally, our data indicate no direct effects of 2-DG treatment to *C. difficile* growth in vitro. Therefore, this, together with the fact that *C. difficile* displays high metabolic flexibility to adapt to distinctive nutrient environments^48^ suggests that the beneficial effects observed upon 2-DG administration in vivo result from host metabolic modulation, and not from the direct interaction with the bacterium. Mitochondrial metabolism is crucial for regeneration and healing of the epithelial barrier. This suggests that preventing the upregulated glycolytic shift during infection preserves the mitochondrial dominant state, with increased epithelial barrier integrity, reduced inflammation and preventing translocation of bacterial strains and the subsequent severe disease, sepsis, and death.

The gut microbiome and the host display a symbiotic relationship required for the maintenance of mucosal homeostasis^49^. Short-chain fatty acids (SCFA), and particularly butyrate, produced by colonic anaerobic bacteria are taken up by intestinal epithelial cells and used as the main fuel for OXPHOS^33^. The high consumption of oxygen by differentiated colonocytes, that mainly rely on oxidative phosphorylation, maintains a very low hypoxic state at the apical surface of the epithelium, shaping the composition of gut commensal bacteria^18^. In addition to the metabolic role, SCFA, and particularly butyrate, also enhance the epithelial barrier function by increasing mucus production^50^ and by promoting formation of tight junctions, through AMPK activation and HIF stabilization^51–53^. Importantly, SCFA inhibit *C. difficile* growth^54, 55^. Upon the shift of colonic metabolism into high glycolytic rates, as observed in mucosal tissues upon CDI, the oxygen consumption decreases and secreted metabolic byproducts shift, allowing the growth of facultative anaerobic bacteria and causing further gut microbiome dysbiosis^18^. Restoration of gut bacterial diversity and composition through fecal microbiome transplantation has shown high efficacy rates^56^, however, the issued FDA safety warning urge the development of alternatives. The modulation of host metabolism through inhibition of glycolysis also results in decreased expression of antimicrobial peptides (AMPs). Thus, the restoration of the metabolic balance in colonocytes upon CDI rescues the homeostatic hypoxic state of the colonic lumen. This together with the downregulation of AMPs may promote the proliferation of commensal obligate anerobic species that confer resistance to *C. difficile* infection and promote epithelium barrier integrity, resulting in lower severity and faster recovery.

We report for the first-time novel systems-wide and mechanistic evidence on the critical role of immunometabolic reprogramming in the mucosal host responses to CDI. Modulation of host cell metabolism by inhibition of glycolysis protects from mortality while suppressing the induction of exacerbated mucosal inflammatory responses and enhancing epithelial barrier function. The results from our studies provide novel evidence of the therapeutic potential of modulating host immunometabolic responses during infection and postulates therapeutic interventions at the intersection of immunity and metabolism as promising antimicrobial-free therapeutic approaches for the treatment of CDI.

## Materials and methods

### Ethic statement and animal housing

C57Bl/6 wild-type (WT) mice were bred and housed in ventilated racks, in a 12:12 light cycle and with *at libitum* access to food and water. Mice were euthanized through CO_2_ narcosis followed by secondary cervical dislocation. If severe signs of disease were present, mice were euthanized before scheduled time points. All experimental procedures performed were approved by the NIMML Institutional Animal Care and Use Committee (IACUC), met or exceeded requirements of the Public Health Service/National Institutes of Health and Animal Welfare Act and were conducted according to the approved guidelines and regulations. This study is reported in accordance with ARRIVE guidelines.

### Animal model of *Clostridioides difficile* infection (CDI)

Before *C. difficile* challenge, C57Bl/6 WT mice were treated with an oral antibiotic cocktail of colistin (4.2 mg/kg), gentamycin (3.5 mg/kg), metronidazole (21.5 mg/kg) and vancomycin (4.5 mg/kg) for a period of three days in drinking water. Three days prior to infection, the antibiotic cocktail was replaced by sterile drinking water. One day before the infection, mice were intraperitoneally injected 32 mg/kg of clindamycin. Mice were then challenged with 10^7^ cfu of *C. difficile* VPI 10463 in 100 μL of sterile Brucella Broth. Uninfected mice received 100 μL of sterile Brucella broth. Mice were monitored daily for disease activity (score 0 to 4) and weight loss for a period of 4, 8 or 10 days. For in vivo validation studies, mice were administered 500 mg/kg/day of 2-Deoxy-d-glucose (2-DG) or vehicle in 200 μL of Phosphate buffer-saline (PBS) through orogastric gavage daily, starting 1 day post infection (dpi). 2-DG (SigmaAldrich) was initially resuspended in dimethyl sulfoxide and further diluted in 1× PBS or cell culture media for in vivo or in vitro studies, respectively.

### In vitro TcdA and TcdB challenge

T84 (ATCC CCL-248) cells cultured in DMEM:F-12 Medium supplemented with 5% FBS and 1% Penicillin/Streptavidin, were plated, and upon reach of confluence, treated with 100 or 250 ng/mL of *C. difficile* toxin A (TcdA) or toxin B (TcdB) (R&D systems) and 2-DG (Sigma-aldrich) at 10 mM or vehicle. 24 h post challenge, cells were collected.

### RNA isolation and targeted gene expression analysis

Total RNA from colon of infected and uninfected mice and harvested T84 cells was extracted using the QIAGEN RNAeasy mini kit (#74106) following manufacturer’s instructions. The BioRad iScript cDNA synthesis kit (#1708891) was used to produce the complementary DNA. Standard curves were generated from a serial dilution of gene-specific amplicons previously produced through polymerase chain reaction (PCR) and purified through QIAGEN MinElute PCR Purification kit (#28004). Total gene expression was assessed through quantitative real-time PCR using the Bio-Rad SybrGREEN mastermix and the CFX96 Bio-Rad Real Time System. Gene expression of target genes is displayed as the normalized values to beta-actin expression. A list of the qRT-PCR primers utilized in this study can be found in Supplementary Table 1.

### Global transcriptome sequencing and bioinformatics analysis

Colonic RNA isolated form *C. difficile*- infected and non-infected mice collected at dpi 0, 3, 4, 5, 8 and 10 was sequenced using Illumina NovaSeq 6000. TapeStation RNA Sample QC was conducted prior to sequencing (RIN values > 8, besides 6 samples between 7.3 and 7.8, and 1 sample at 6.5). Libraries for RNA-seq were prepared using the SeqMatic TailorMix Directional mRNA library preparation kit. Sequencing reads were filtered to remove adapter sequences and low-quality terminal bases via cutadapt. Filtered reads were aligned and quantified to the Ensembl mouse reference genome (GRCm38) using Salmon. Normalization of counts and differential expression analysis was conducted by DESeq2^57^. A linear discriminant analysis was performed on the gene expression data using the sklearn package for Python. Gene set enrichment analysis (GSEA) and visualization of the GSEA results were performed using the GSEApy package in Python^58^. A further statistical analysis was conducted to identify the number of genes that were differentially expressed due to the time and infection variables of the study. A likelihood-ratio test (Wilks test) was performed using the DESeq2 package in R with a significance threshold of p < 0.05 for the time and infection variables. Agglomerative hierarchical clustering was performed using Euclidean distance as a measure of similarity between samples. The clustering analysis was conducted with the seaborn package in Python. A separate agglomerative hierarchical clustering analysis was also used to compare the gene expression of each gene for each sample within three pathways (TCA cycle, Oxidative Phosphorylation, and Glycolysis/Gluconeogenesis). The gene sets utilized for the clustering of each metabolic pathway were obtained from the KEGG^59–61^ pathways TCA cycle, Oxidative Phosphorylation, and Glycolysis/Gluconeogenesis. Heatmap graphs were created using matplotlib.

### Metabolic profile analysis using the M^2^ pipeline

The previously developed Modeling Metabolism (M^2^) Pipeline^23^ was utilized. Briefly, metabolic enzyme concentrations were estimated from the 10-day time course RNA-seq study of *C. difficile* infection by normalizing TPM values to estimated total protein contents of human cells. The estimated protein concentrations were used to fit polynomial equations using the Scipy package in Python, which determined the algebraic expression for polynomial equations ranging from rank 0 to 5 as was necessary for inclusion in the differential equation model. The fitted equations were transformed to reference simulation time so they could be included in the Systems Biology Markup Language (SBML) compliant differential equations model of metabolic networks^62^. The system of differential equations included 131 species with 57 reactions, which included the pathways: glycolysis, gluconeogenesis, pentose phosphate pathway, tricarboxylic acid cycle, oxidative phosphorylation, fatty acid oxidation, glutaminolysis, and the malate-aspartate shuttle. The rate laws used in the model to calculate the flux of reactions were Michaelis Menten^63^, ping-pong bi-bi, and Hill-like equations^64^. The model was solved using PycoTools^65^, with a simulation time of 240 h. The resulting time courses were plotted using the Matplotlib package in python.

### Flow cytometry

Colons were excised, rinsed in 1× PBS and incubated with RPMI supplemented with collagenase (300 U/mL) and DNAse (50 U/mL) 1 h at 37 °C stirring. Samples were filtered and the immune cell fraction was enriched through a percoll gradient. Cells were washed and plated for immunophenotyping. Samples were incubated with Fc-Block for 10 min, followed by a 20-min incubation with a mix of fluorochrome-conjugated antibodies against extracellular markers (CD45, CD4, CD3, CD8, NK1.1 CD19, F4/80, MHCII, CD11b, Gr1, CD11c, Ly6c). Cells were then fixed and permeabilized in preparation for an additional 20-min incubation in a mix of fluorochrome-conjugated antibodies for the detection of intracellular TNFa. Samples were analyzed in a BD FACS Celesta instrument and the statistical and plot analyses of acquired events were performed using FACSDIVA software. The gating schemes for the flow cytometry analysis are included in Supplementary Fig. 7. A list of the fluorochrome-conjugated antibodies for flow cytometry staining utilized in this study can be found in Supplementary Table 2.

### Quantification of *C. difficile *in vitro

*C. difficile* VPI 10463 was inoculated in anaerobic chopped meat media tubes (Hardy Diagnostics) injected with vancomycin (10 μg/mL), 2-DG (1 μg/mL, 10 μg/mL or 100 μg/mL), or untreated, and incubated for 8 h at 37 °C. After incubation, samples were serially diluted and plated in *C. difficile* agar base (Oxoid) plates supplemented with 7% laked horse blood (Lampire Biological Laboratories) and *C. difficile* selective supplement (Oxoid) and incubated anaerobically. Colonies were counted 3 days after culture.

### Assessment of HK activity

Colons from infected and uninfected mice were excised at day 3, 5 and 8 post infection and homogenized using a MP Biomedical FastPrep-24 bead beating grinder (20 s, 4 m/s, twice). Samples were centrifuged for 10 min 13,000×*g* at 4 °C, and the supernatants were used to quantify enzymatic activity using the Sigma-Aldrich HK colorimetric assay kit (#MAK091) following manufacturer’s instructions. In order to compare the HK activity between samples, the initial enzymatic activity obtained from each sample was normalized to the sample tissue weight. Displayed results have been normalized to the uninfected group in each time point.

### Transepithelial electrical resistance (TEER)

T84 cells were plated in the apical surface of 0.4 mm transwell inserts and cultured for five days. Baseline TEER was measured in duplicate in each insert at time 0 using the Millipore Sigma Millicel ERS-2 Epithelial Vol-Ohm meter. Only wells displaying TEER higher than 190 ohms were included. Cells were challenged with 250 ng/mL of TcdA or TcdB (R&D systems) and treated with 2-DG (Sigma-Aldrich) at 10 mM or vehicle. 24 h post challenge TEER was measured. Results are displayed as percentage of change at 24 h versus the baseline.

### Statistical analysis

Data is expressed as the mean and standard error of the mean represented in error bars. To determine statistical significance of the experimental data generated, analysis of variance (ANOVA) was performed in R. Significance was identified with an asterisk (*) and considered at *P-value* ≤ 0.05.

### Supplementary Information

Supplementary Information.

## Data Availability

The datasets generated and analyzed during the current study are available in NCBI’s GEO database, Accession Number GSE237574.
