# Supplementary material for: Modulation of colonic immunometabolic responses during Clostridioides difficile infection ameliorates disease severity and inflammation
Source: Sci Rep. 2023 Sep 7;13:14708. doi: 10.1038/s41598-023-41847-2 (PMC10485029; doi:10.1038/s41598-023-41847-2)
Supplement: Supplementary file 1 — Supplementary Information. [file 41598_2023_41847_MOESM1_ESM.pdf]

Modulation of colonic immunometabolic responses during *Clostridioides difficile* infection ameliorates disease severity and inflammation

Nuria Tubau-Juni[1], Josep Bassaganya-Riera[1], Andrew J. Leber[1], Sameeksha S. Alva[1], Ryan Baker[1] and Raquel Hontecillas[1*]

[1] NIMML Institute, Blacksburg, VA 24060, USA.

Correspondence: Dr. Raquel Hontecillas, NIMML Institute, Blacksburg, VA 24060, USA. E-mail: rmagarzo@nimml.org.

Supplementary information

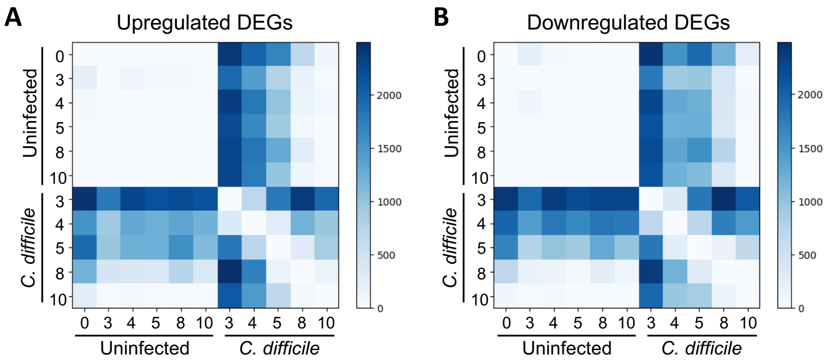

Supplementary Figure 1. Additional DEGs analysis from global transcriptomics analysis from 10-day time course *C. difficile* infection (CDI) study from colonic samples. Heatmap displaying the number of DEGs from sample-to-sample analysis for upregulated (**A**) and downregulated (**B**) DEGS. Plots represent the number of DEGs from the comparison of samples in the X axes compared to the samples in the Y axes.

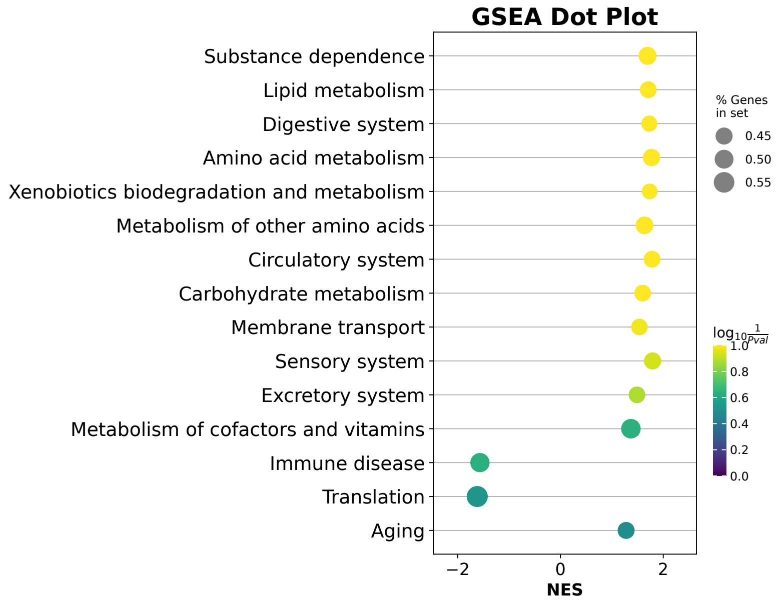

Supplementary Figure 2. Gene set enrichment analysis (GSEA) from bulk RNAseq dataset for KEGG pathway groups. The dot plot displays the enrichment scores of the most statistically significant enriched pathway groups, with each dot indicating the percentage of genes enriched based on dot size, the enrichment score based on the position, and the corresponding p-value based on the color. The plot indicates high levels of enrichment of groups of metabolic pathways.

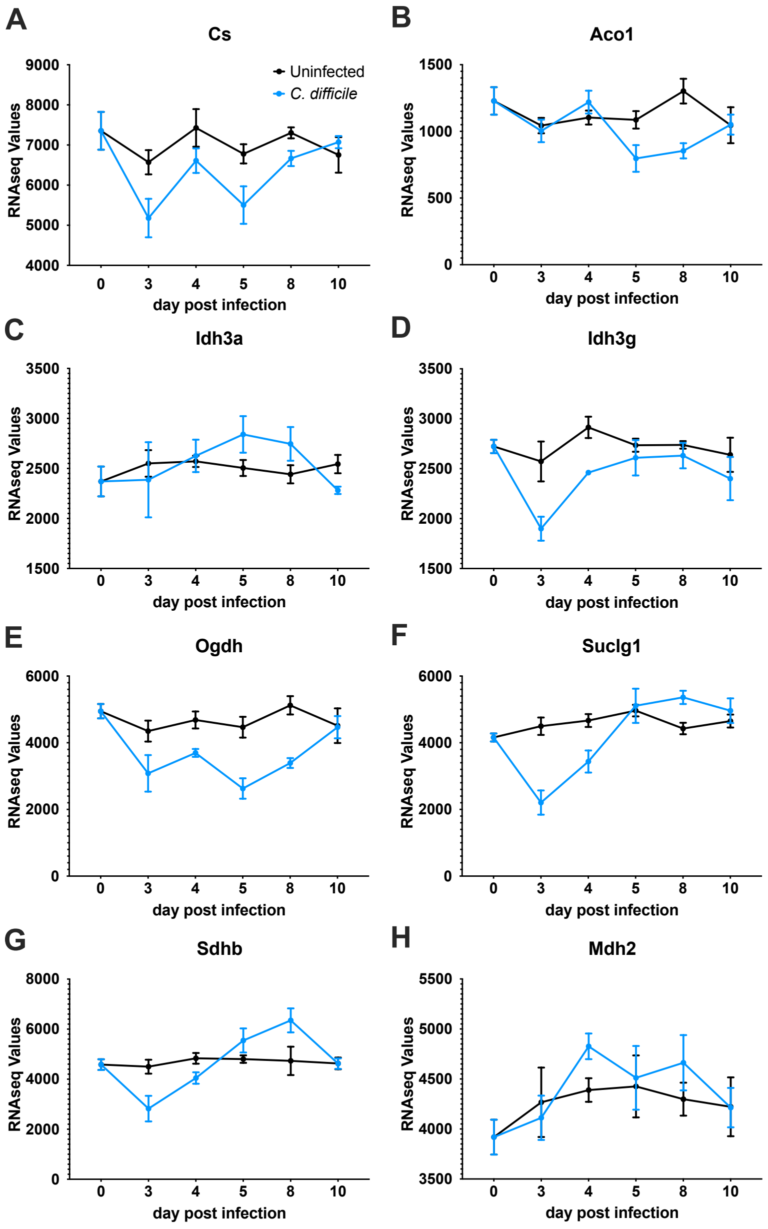

Supplementary Figure 3. Colonic expression of TCA cycle genes in the 10-day time course RNA-seq study of CDI. RNA-seq values from colonic TCA Cycle genes Citrate synthetase (**A**, CS), Acotinase 1 (**B**, Aco1), Isocitrate dehydrogenase (NAD(+)) 3 Catalytic Subunit alpha subunit (**C**, Idh3a), Isocitrate dehydrogenase (NAD(+)) 3 Gamma (**D**, Idh3g), Oxoglutarate dehydrogenase (**E**, Ogdh), Succinate-CoA Ligase GDP/ADP-Forming Subunit Alpha (**F**, Suclg1), Succinate dehydrogenase Complex Iron Sulfur Subunit B (**G**, Sdhb), and Malate Dehydrogenase 2 (**H**, Mdh2) from uninfected and *C.difficile*-infected samples of the 10-day RNA-seq dataset.

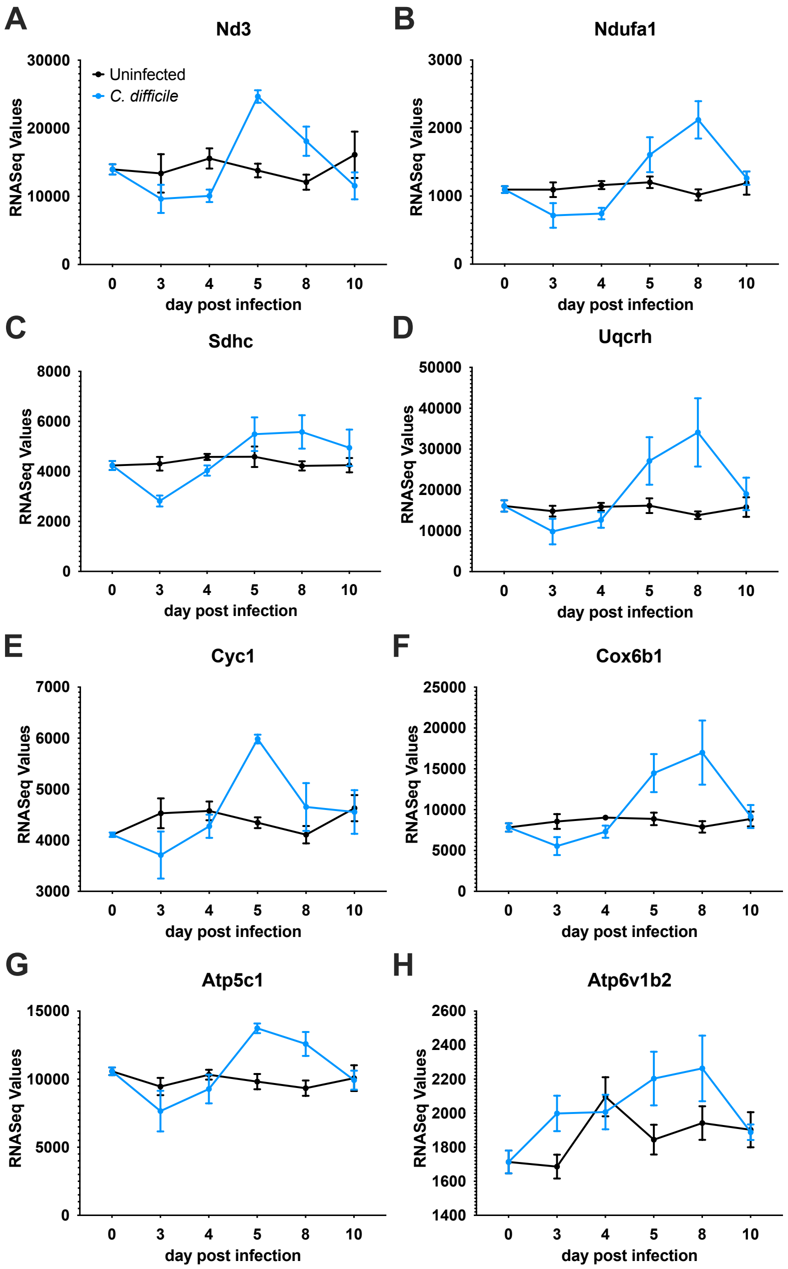

Supplementary Figure 4. Colonic expression of oxidative phosphorylation genes in the 10-day time course RNA-seq study of CDI. RNA-seq values from colonic oxidative phosphorylation genes NADH dehydrogenase subunit 3 (**A**, Nd3), NADH:Ubiquinone Oxidoreductase Subunit A1 (**B**, Ndufa1), Succinate dehydrogenase complex C (**C**, Sdhc), Ubiquinol-Cytochrome C Reductase, Complex III Subunit VIII (**D**, Uqcrh), Cytochrome C1 (**E**, Cyc1), Cytochrome C Oxidase Subunit 6B1 (**F**, Cox6b1), ATP Synthase F1 Subunit Gamma (**G**, Atp5c1), ATPase H+ transporting V1 subunit B1 (**H**, Atp6v1b2) from uninfected and *C.difficile*-infected samples of the 10-day RNA-seq dataset.

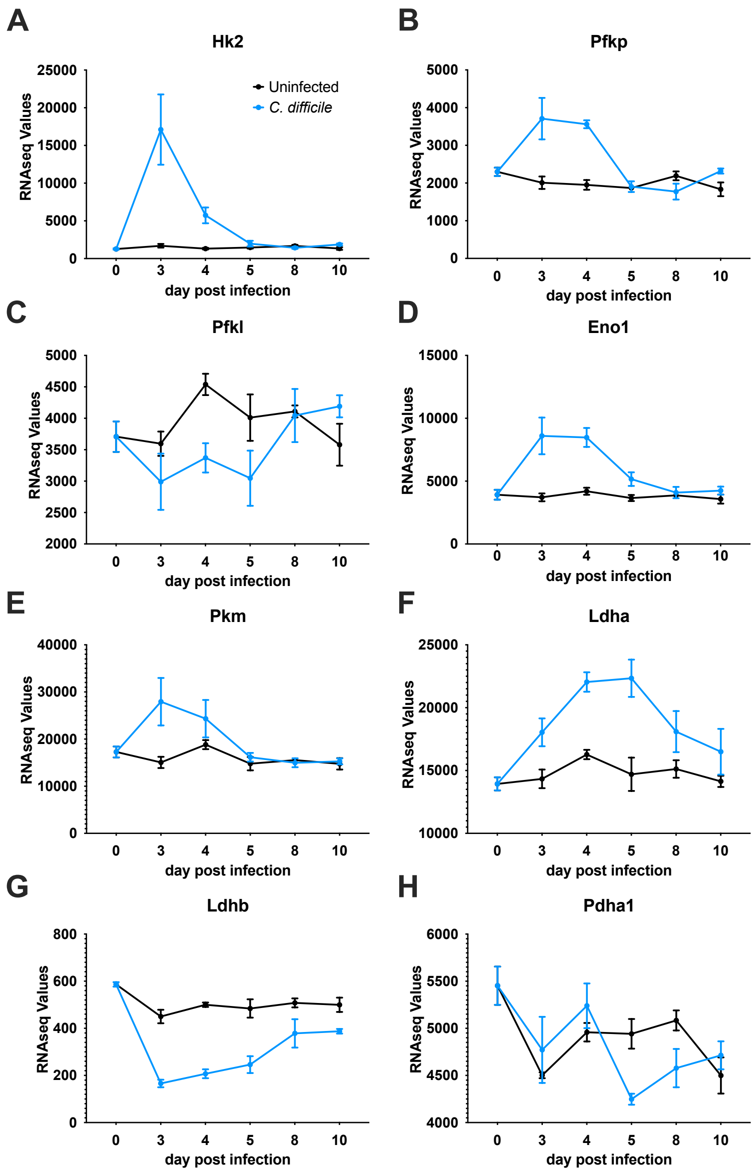

Supplementary Figure 5. Colonic expression of glycolysis and gluconeogenesis genes in the 10-day time course RNA-seq study of CDI. RNA-seq values from colonic glycolytic genes Hexokinase 2 (**A**, Hk2), Phosphofructokinase, platelet (**B**, Pfkp), Phosphofructokinase, Liver type (**C**, Pfkl), Enolase 1 (**D**, Eno1), Pyruvate kinase M1/2 (**E**, Pkm), Lactate dehydrogenase A (**F**, Ldha), Lactate dehydrogenase B (**G**, Ldhb) and Pyruvate dehydrogenase A 1 (**H**, Pdha1) from uninfected and *C.difficile*-infected samples of the 10-day RNA-seq dataset.

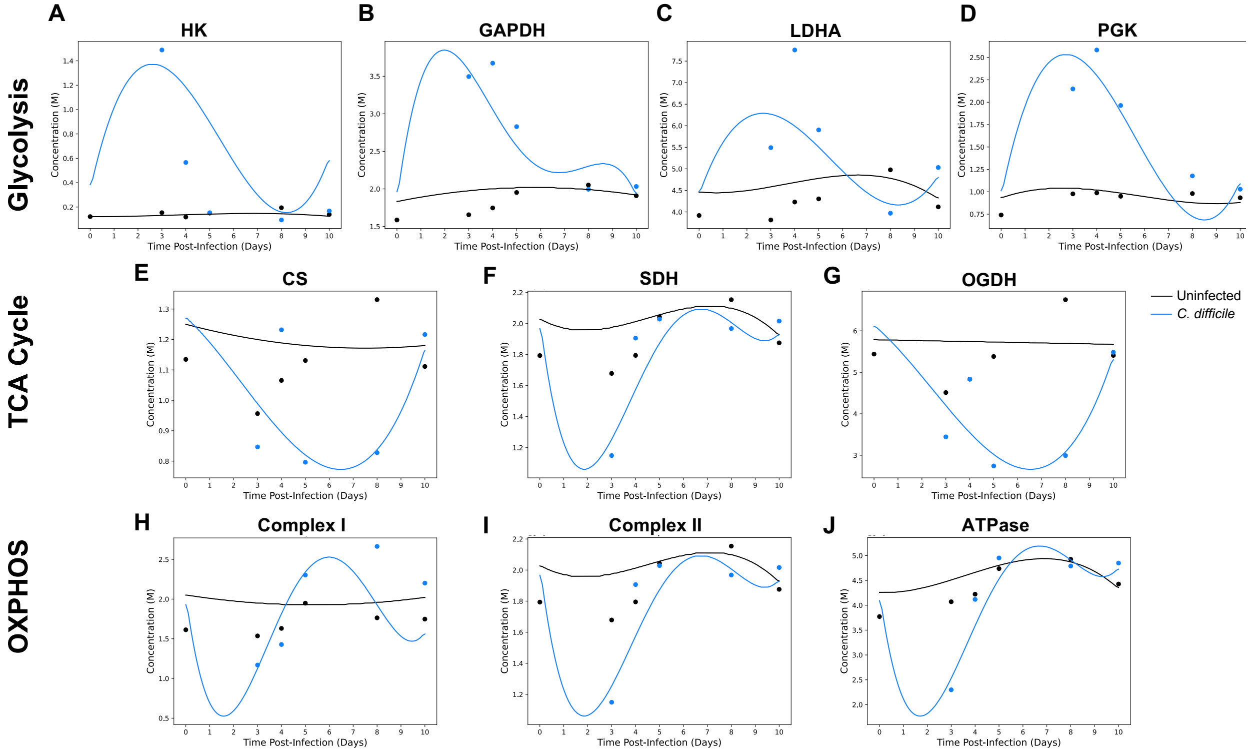

Supplementary Figure 6. Fitting of M^2 pipeline simulations to colonic host responses to CDI with estimated protein concentrations derived from experimental data points. Simulated concentration of enzymes and protein complexes hexokinase (HK, **A**), glyceraldehyde-3-phosphate dehydrogenase (GAPDH, **B**), lactate dehydrogenase A (LDHA, **C**), phosphoglycerate kinase (PGK, **D**), citrate synthase (CS, **E**), succinate dehydrogenase (SDH, **F**), oxoglutarate dehydrogenase (OGDH, **G**), oxidative phosphorylation complex I (**H**), complex II (**I**), and complex V or ATPase (**J**). Lines represent simulation results, while dots represent the average estimated protein concentration values derived from experimental data.

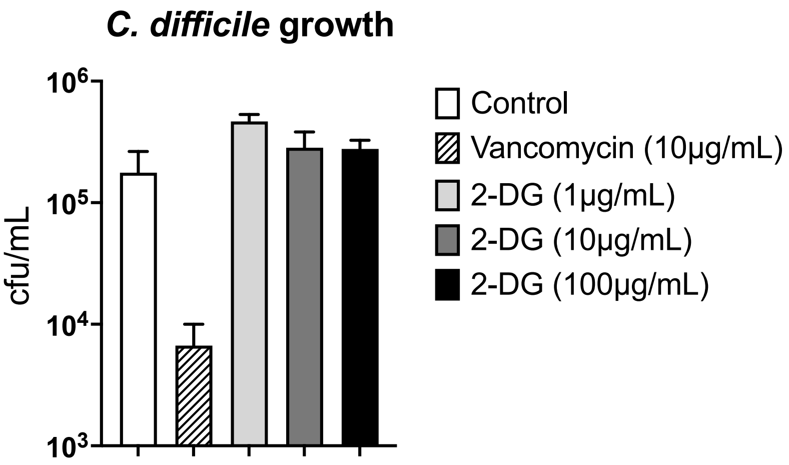

Supplementary Figure 7. Effects of 2-Deoxy-D-glucose (2-DG) on *C. difficile* growth *in vitro*. *C. difficile* VPI10463 was inoculated in anaerobic chopped meat media tubes treated with vancomycin (10 μg/mL) or 2-DG (1 μg/mL, 10 μg/mL or 100 μg/mL) or untreated (control). Tubes were incubated for 8 h at 37 C and bacterial loads were assessed. Results are expressed as colony forming units (cfu) per mL. Data expressed as mean ± SEM (n = 3).

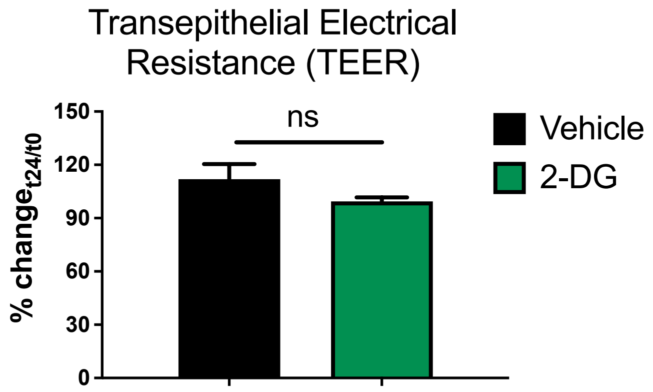

Supplementary Figure 8. Transepithelial Electrical Resistance of T84 cells treated with 2-DG *in vitro*. T84 cells were treated with vehicle or 2-DG (10 mM) and cultured for 24 h. Transepithelial electrical resistance (TEER) was quantified. Data expressed as mean ± SEM (n = 5).

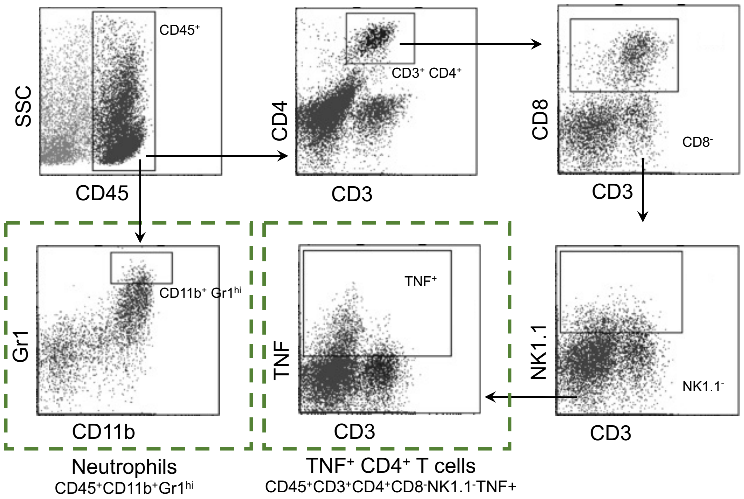

Supplementary Figure 9. Gating schemes for flow cytometry analysis. Gating strategy for TNF+ CD4+ T cells (CD45+ CD3+ CD4+ CD8- NK1.1- TNF+) and Neutrophils (CD11b+ Gr1^{hi}) using the BD Biosciences FACSDiva software.

Gene	Species	Forward Primer	Reverse Primer
β-Actin	Mouse	CCGAGGCATTGCTGCCAGG G	TGGAAGGTCGACAGTGAGGC
Hk2	Mouse	GCATCGAGAGAACTCGACCAAAC	GACAGCGTGCATCTCGTGTAAA
Eno1	Mouse	TCTGGCAAGTATGACCTGGACT	ATGGACACCACTGGGTAGTTCT
Ldhb	Mouse	GCTCAACCTGGTGCAGAGAAAT	TGGGTTGGAAACCACGATGATG
Pdha	Mouse	CAAAGTTTGCGGCTGCCTATTG	AGCTTACTCCAGGGTCACTCAT
Cs	Mouse	CCAACCAATCTGCACCCTATGT	TCCCAGTACTTAGCTCGGTTCA
Ogdh	Mouse	CAGGGTAGGAACAGAACCCTATGT	GCTTGGCCCTAGCAAGATGAAA
Suclg1	Mouse	CAGGTCCGGTACTCTGACTTATGA	GGTCACCTCCAATGCCAATACA
Sdhb	Mouse	GCTGCCACACCATCATGAACT	TCCTTGTAGGTCGCCATCATCT
Ndufa1	Mouse	CAATCGCTACTATGTGTCCAAGGG	GCTGCATAGCCTTCTAACAGGAAC
Uqcrh	Mouse	AGGACGAACGAAAGATGCTCAC	ACACACTTCTCCAGCTGTTCAC
Cox1	Mouse	AGGCTTCACCCTAGATGACACA	TTCCTGAAAGGCCCAGGAAATG
Atp6v1b2	Mouse	GGCTTGAGCTCTTGGCTGTATT	AACTGGAAGGTGGAATGCACAG
Ldha	Mouse	ACGAGGTGATCAAGCTGAAAGG	CACCCGCCTAAGGTTCTTCATT
Il1b	Mouse	TGAAGAAGAGCCCATCCTCTGT	TGTTCATCTCGGAGCCTGTAGT
Il6	Mouse	AAGGAGTGGCTAAGGACCAAGA	GGTTTGCCGAGTAGATCTCAAAGTG
Mcp1	Mouse	ACCTCTCTCTTGAGCTTGGTGA	TGATCCCAATGAGTAGGCTGGA
S100A9	Mouse	CGACACCTTCCATCAATACTCT	TTGCCAACTGTCCTTCCA
S100A8	Mouse	GAGGGCATGGTGATTTCCTTGT	CGTGACAATGCCGTCTGAACT
Ocln	Mouse	CCACCTCCTTACAGACCTGATGAA	AAAGAGTACGCTGGCTGAGAGA
β-Actin	Human	TGGACATCCGCAAAGACCTGTA	AGTGATCTCCTTCTGCATCCTGTC
Il1b	Human	CCACAGACCTTCCAGGAGAATGA	ACAGGTGCATCGTGCACATAAG
Tnfa	Human	GACAAGCCTGTAGCCCATGTT	AGCTGGTTATCTCTCAGCTCCA
Il8	Human	ACACTGCGCCAACACAGAAA	AAACTTCTCCACAACCCTCTGC
Ocln	Human	CCATTAACTTCGCCTGTGGATGAC	CTTCCCTTTGCAGGTGCTCTTT
ZO-1	Human	GGGAGCACATGGTGAAGGTAATTC	CTTCTCGGTTTGGTGGTCTGAAAG
JAM1	Human	TAGTGCCCGAAGTGAAGGAGAA	GGTAGCACCTGAGTAAGGCAAATG

Supplementary Table 1. qRT-PCR primers utilized in this study.

Antibody	Vendor	Catalog number	Clone number	Fluorochrome	RRID
CD45	Invitrogen	47-0451-82	30-F11	APC-eFluor™ 780	AB_1548781
CD3	BD Biosciences	562600	145-2C11	BD Horizon™ BV421	AB_11153670
CD4	BD Biosciences	563151	RM4-5	BD Horizon™ BV605	AB_2687549
CD8	BD Biosciences	563332	53-6.7	BD Horizon™ BV786	AB_2721167
NK1.1	Invitrogen	64-5941-82	PK136	Super Bright™ 645	AB_2662737
TNF	Invitrogen	17-7321-82	MP6-XT22	APC	AB_469508
CD11b	BD Biosciences	557960	M1/70	APC-R700	AB_396960
Gr1	Invitrogen	64-5931-82	RB6-8C5	Super Bright™ 645	AB_2662805

Supplementary Table 2. Fluorochrome-conjugated anti-mouse antibodies for flow cytometry staining utilized in this study.
